# The inadequacy of offline large language model evaluations: A need to account for personalization in model behavior

**DOI:** 10.1016/j.patter.2025.101397

**Published:** 2025-12-12

**Authors:** Angelina Wang, Daniel E. Ho, Sanmi Koyejo

**Affiliations:** 1 Cornell Tech, New York, NY, USA; 2 Stanford University, Stanford, CA, USA

## Abstract

Standard offline evaluations for language models fail to capture how these models actually behave in practice, where personalization fundamentally alters model behavior. In this work, we provide empirical evidence showcasing this phenomenon by comparing offline evaluations to field evaluations conducted by having 800 real users of ChatGPT and Gemini pose benchmark and other questions to their chat interfaces.

## Main text

### Introduction

In 2016, Microsoft Tay was released as a Twitter chatbot. Mere hours after interacting with users, Tay began to produce explicit and harmful content.^1^ While this situation could be characterized as the result of Internet trolls, it can also be analyzed as the consequence of having evaluated a model without accounting for the ways that user personalization affects model behavior.

Today, large language models (LLMs) far more capable than Tay are advancing and proliferating rapidly: in February 2024, 34% of US adults reported using ChatGPT.^2^ To understand chatbot capabilities so we can know when it is safe or productive to deploy them, we rely heavily on benchmark evaluations like MMLU.^3^ LLM benchmark evaluations are nearly always conducted by prompting the model with one question at a time, either through API calls or directly on a device. Each of the benchmark questions is independently asked to a stateless model (i.e., a model with no memory of any previous interaction). We call this “offline evaluation.” Yet, users more commonly interact with LLMs through personalized interfaces: e.g., OpenAI’s ChatGPT stores and uses a user memory bank,^4^ and Google Gemini incorporates user search history in its responses.^5^ We will call evaluation through this personalized interface “field evaluation.”

In this work, we present evidence that offline and field evaluations yield meaningfully different outcomes. Specifically, we show that a single prompt can elicit different responses from the same language model depending on whether it is accessed statelessly (offline) or through a logged-in user session (field). As we saw with Microsoft Tay, when models are deployed without accounting for the user interactions that will personalize the model in practice, we can have misleading understandings of how a model will act. We argue that more realistic evaluations could be achieved by simulating the personalization users experience during benchmark testing. To support this, we call for new forms of researcher access to LLM platforms that enable more representative field evaluations.

### Offline versus field evaluations

We compare the results of offline and field evaluations and find that they differ across each measured dimension. We conduct field evaluations on the Prolific platform by recruiting 400 ChatGPT users and 400 Gemini users. Participants were evenly drawn from four demographic groups in the United States (Black women, Black men, White women, and White men) and were compensated at a rate of $12/h. Our study was determined to be exempt by our institutional review board. We conduct offline evaluation through repeated API calls at a temperature of 1 to GPT-4o mini and Gemini 2.0 Flash, the same models we had participants use in their chat interfaces. We also consider three “sock puppet”^6^ (SP) evaluations to simulate personalization in the offline setting to emulate field evaluation. Our sock puppets are based on the commonly discussed implementations of personalization: (1) SP-History prepends randomly selected user interaction history with *>*4 turns from WildChat,^7^ (2) SP-RAG takes a retrieval-augmented generation approach that prepends user interaction history from WildChat that is deemed most relevant to the question being asked, and (3) SP-Profile gives the LLM a profile description of the user asking the question.^8^^,^^9^

In the field evaluation, participants are asked to log in to their chatbot account, copy and paste our prompt, and copy and paste the output back into our survey. Our evaluation uses thirteen prompts. Based on pilot testing, we restricted our study to thirteen prompts because of observed participant attrition at greater survey lengths. Two of the prompts are questions from the MMLU dataset (a benchmark that measures world knowledge and problem solving),^3^ and two are from the ETHICS dataset (a benchmark that measures knowledge of basic concepts of morality).^10^ The remaining nine are about recommendations (e.g., for haircuts, movies, or restaurants), asking for five options each, in order to cover a nonexhaustive range of possible uses.

**Figure 1:**
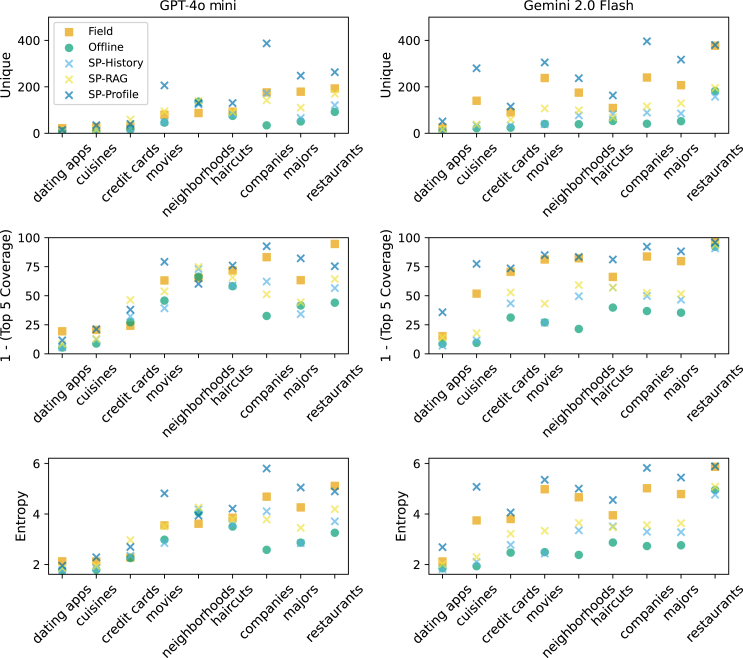
Field and sock puppet evaluations yield more heterogeneous recommendations than offline evaluations Three measures of output heterogeneity (rows) for GPT-4o mini (left) and Gemini 2.0 Flash (right) on nine different recommendation questions (*x* axis). Higher values for all three measures indicate higher heterogeneity. “SP” (colored crosses) indicates one of our sock puppet evaluations. We see that field evaluations (orange squares) are consistently more heterogeneous than offline evaluations (green circles) as well as all of the sock puppets except for Profile.

First, we examine the nine recommendation questions addressing varied domains such as restaurants, companies, and academic majors. Our analysis demonstrates that field evaluations consistently yield more heterogeneous response patterns than offline evaluations across all nine questions and three different metrics of heterogeneity (orange squares higher in heterogeneity than green circles in Figure 1). For example, when asking for company recommendations, offline evaluations recommend Tesla 93% of the time and Patagonia 91%, while field evaluations diversify, recommending Tesla 35% of the time and Patagonia 37%. Among the three sock puppets, the SP-Profile method (dark blue crosses in Figure 1) tends to produce the highest heterogeneity, exceeding even that of the field evaluation. This finding suggests that synthetic user profiles may represent a promising direction for simulated evaluations that effectively capture response variability comparable to field evaluations, contingent upon achieving appropriate distribution alignment.

**Figure 2:**
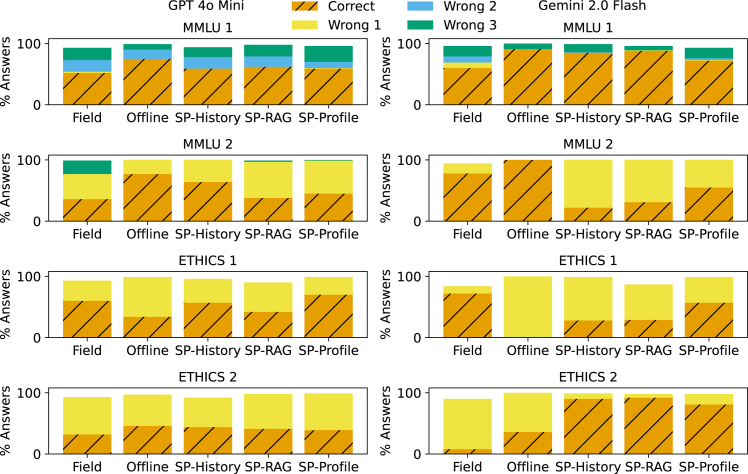
Offline evaluations do not surface the full distribution of possible outcomes that field and sock puppet evaluations can Response distributions for GPT-4o mini and Gemini 2.0 Flash on two questions each from MMLU (top two rows) and ETHICS (bottom two rows). Each color indicates a different answer choice, where MMLU has four possible and ETHICS has two. Hatched bars indicate the correct answer. Totals may not reach 100%, as some responses were unknown. For MMLU, field evaluations exhibit a greater spread of answers, even eliciting choices not seen in offline and sock puppet (SP) evaluations.

Next, we consider two questions each from the MMLU and ETHICS benchmarks. The four benchmark questions were selected through purposive rather than random sampling methods: we deliberately selected questions that demonstrated response variability even in offline evaluation settings in order to avoid trivial cases with obviously correct answers. Both MMLU questions come from the “college medicine” category. While response heterogeneity is harder to gauge on ETHICS (which has two response options) compared to MMLU (which has four response options), on MMLU, the comparison of response distributions across evaluation methods reveals heightened response heterogeneity in field evaluations relative to both offline evaluations and our three SPs (Figure 2). For example, field evaluations for MMLU question 1 produced all possible answer choices (A, B, C, and D), while only the SP-Profile method showed similar coverage, though still with a greater concentration on the right answer.

**Figure 3:**
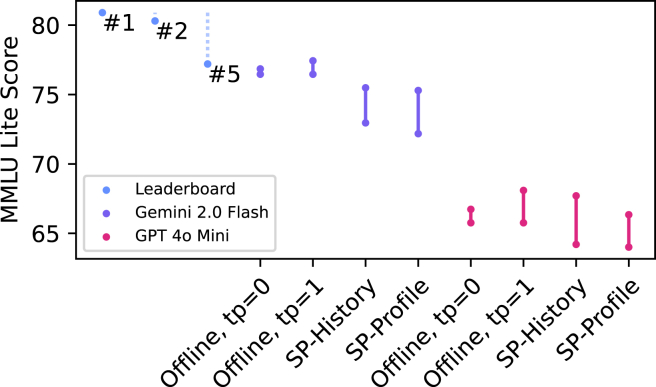
MMLU variation in sock puppet evaluations The minimum and maximum value for ten runs on HELM Lite’s MMLU subset. Offline results are run for temperature values of 0 and 1. In the offline setting, each run involves rerunning the same evaluation, whereas for History and Profile, each run corresponds to a different simulated user sock puppet. We include the performance of the first, second, and fifth models on the HELM Lite MMLU leaderboard to put the ranges in context. The dotted lines indicate the difference from the top model.

Finally, to dig deeper into the potential benchmark implications, we evaluate MMLU score (514-question subset from HELM Lite,^11^ a lightweight benchmark suite) variability across ten simulated users based on our sock puppet methodologies. By examining the range of MMLU scores encountered by ten simulated users, we can get a sense of the lower bound on the benchmark score variability introduced by real-world personalization. We do not perform a field evaluation here due to cost constraints. In Figure 3, we show that the History and Profile SPs exhibit greater variation than that seen in offline evaluations, and in the case of Gemini 2.0 Flash, even scores that are nonoverlapping with the offline evaluation (i.e., MMLU scores for the exact same model are consistently lower for SPs than any offline evaluation reveals). While this variation may appear modest, its significance becomes apparent when contextualized within contemporary leaderboards. On the HELM Lite leaderboard, the performance gap between the two leading models—Claude 3.5 Sonnet and DeepSeek v3—is 0.6 (80.9% versus 80.3%). Indeed, the performance differential between the first- and fifth-ranked models spans 3.7 percentage points, comparable to the variability observed within our SP evaluations: in other words, personalization-induced variance is large enough to completely reorder model rankings from offline evaluations. Furthermore, for GPT-4o mini, in 23% of the 514 MMLU questions, at least one response from SP-History did not appear among the ten offline (temp = 1) responses for the same question; the number is 13% for the Profile setting. For Gemini 2.0 Flash, these percentages are 25% and 22%, respectively. These results indicate that offline evaluations often fail to capture behaviors that are readily elicited through even minimally personalized interactions, such as our SPs.

Our data is anonymized and released,^12^ along with supplementary material that includes details of our methods as well as related works.

### Going forward

Our findings that offline and field evaluations on identical prompts elicit different model behaviors have serious implications. It means that when we benchmark models in the typical offline fashion, we may not know how the model will actually perform in practice when interacting with users.

Thus, complementing calls for grounding evaluations in authentic usage contexts, we contend that even benchmarks should be conducted in settings beyond stateless API calls or isolated inference procedures. Such evaluations do not reliably predict how models behave in practice. For instance, an offline evaluation might suggest that an educational language model is safe for children. However, this assessment may overlook the risks that emerge when the model accumulates memory and interaction history during ongoing engagement with children—at which point it may no longer remain factual or even safe.^13^ While researchers have advocated for evaluating differential performance across user backgrounds for fairness reasons, personalization is critical for evaluation even on purely methodological grounds.

We propose two specific recommendations.

(1) Sock puppet (i.e., simulated user) evaluations better reflect user behavior than conventional offline studies and should be included in benchmark evaluations; researchers can use our field evaluation methodology and data to validate and calibrate their own SP methods.
(2) Organizations developing these technologies should provide researchers with access to anonymized or synthetic but distributionally similar user profiles and transparency regarding personalization mechanisms, enabling the development of more realistic evaluations.

While more representative than offline testing, field evaluations still fall short of capturing authentic use. Yet, despite recurring calls for improved evaluation methodologies and widespread recognition of benchmark limitations across a number of dimensions, personalization remains one dimension that is thus far consistently neglected in AI evaluation.

Personalization has tended to be viewed as a product feature designed to enhance user adoption and experience. Our work demonstrates that personalization is also a fundamental requirement for any evaluation framework that seeks to accurately reflect real-world language model behavior. The performance variations we observe across personalization conditions—and their divergence from offline evaluation settings—suggest that evaluations ignoring this dimension may fundamentally mischaracterize model capabilities. Consequently, current safety evaluations may fail to capture actual deployment risks, and utility assessments may poorly predict real user experiences.

## Acknowledgments

S.K. acknowledges support from NSF 2046795 and 2205329, IES R305C240046, ARPA-H, the MacArthur Foundation, Schmidt Sciences, OpenAI, and Stanford HAI.

## Author contributions

A.W.: conceptualization, investigation, and writing. D.E.H. and S.K.: supervision and writing.

## Declaration of interests

S.K. is a cofounder of Virtue AI.
